# Reassessment of *Faxinalipterus minimus*, a purported Triassic pterosaur from southern Brazil with the description of a new taxon

**DOI:** 10.7717/peerj.13276

**Published:** 2022-05-03

**Authors:** Alexander W.A. Kellner, Borja Holgado, Orlando Grillo, Flávio Augusto Pretto, Leonardo Kerber, Felipe Lima Pinheiro, Marina Bento Soares, Cesar Leandro Schultz, Ricardo Tadeu Lopes, Olga Araújo, Rodrigo Temp Müller

**Affiliations:** 1 Laboratório de Sistemática e Tafonomia de Vertebrados Fósseis, Setor de Paleovertebrados, Departamento de Geologia e Paleontologia, Museu Nacional, Universidade Federal do Rio de Janeiro, Rio de Janeiro, Brazil; 2 Institut Català de Paleontologia Miquel Crusafont, Universitat Autònoma de Barcelona, Barcelona, Cataluña, Spain; 3 Centro de Apoio à Pesquisa Paleontológica da Quarta Colônia, Universidade Federal de Santa Maria, São João do Polêsine, Rio Grande do Sul, Brazil; 4 Programa de Pós-Graduação em Biodiversidade Animal, Universidade Federal de Santa Maria, Santa Maria, Rio Grande do Sul, Brazil; 5 Laboratório de Paleobiologia, Universidade Federal do Pampa, São Gabriel, Rio Grande do Sul, Brazil; 6 Departamento de Paleontologia e Estratigrafia, Universidade Federal do Rio Grande do Sul, Porto Alegre, Rio Grande do Sul, Brazil; 7 Laboratório de Instrumentação Nuclear, Programa de Engenharia Nuclear, Universidade Federal do Rio de Janeiro, Rio de Janeiro, Brazil

**Keywords:** Archosauria, Pterosauromorpha, Triassic, Santa Maria Supersequence, Rio Grande do Sul, Brazil

## Abstract

*Faxinalipterus minimus* was originally described as a purported pterosaur from the Late Triassic (early Norian) Caturrita Formation of southern Brazil. Its holotype comprises fragmentary postcranial elements, whereas a partial maxilla was referred to the species. The assignment of *Faxinalipterus minimus* to Pterosauria has been questioned by some studies, but the specimen has never been accessed in detail after its original description. Here we provide a reassessment of *Faxinalipterus minimus* after additional mechanical preparation of the holotype. Our interpretations on the identity of several bones differ from those of the original description, and we found no support favoring pterosaur affinities for the taxon. The maxilla previously referred to *Faxinalipterus minimus* is disassociated from this taxon and referred to a new putative pterosauromorph described here from a partial skull and fragmentary postcranial elements. *Maehary bonapartei* gen. et sp. nov. comes from the same fossiliferous site that yielded *Faxinalipterus minimus*, but the lack of overlapping bones hampers comparisons between the two taxa. Our phylogenetic analysis places *Faxinalipterus minimus* within Lagerpetidae and *Maehary bonapartei* gen. et sp. nov. as the earliest-diverging member of Pterosauromorpha. Furthermore, the peculiar morphology of the new taxon reveals a new dental morphotype for archosaurs, characterized by conical, unserrated crowns, with a pair of apicobasally oriented grooves. These two enigmatic archosaurs expand our knowledge on the Caturrita Formation fauna and reinforce the importance of its beds on the understanding of Late Triassic ecosystems.

## Introduction

In the last two decades, there has been a wealth of new information on Triassic vertebrates from Southern Brazil, mainly due to the systematic collecting efforts carried out by several Brazilian institutions. The top of the Candelária Sequence (Horn et al., 2014) of the Santa Maria Supersequence (Zerfass et al., 2003), corresponding to the Caturrita Formation (*sensu* Andreis, Bossi & Montardo, 1980), early Norian in age (Soares, Schultz & Horn, 2011; Langer, Ramezani & Da-Rosa, 2018), is amongst the units that have demonstrated the richest yielding of fossil vertebrates. These fossils are assigned to the *Riograndia* Assemblage Zone (AZ) (Soares, Schultz & Horn, 2011). Most Caturrita Fm. specimens belong to small-sized vertebrates with fragile skeletal elements, including procolophonids, non-rhynchocephalian lepidosauromorphs, sphenodontians and specialized non-mammaliaform probainognathian cynodonts (*e.g.*, Bonaparte, Ferigolo & Ribeiro, 2001; Bonaparte et al., 2003; Bonaparte et al., 2010; Cisneros & Schultz, 2003; Martinelli et al., 2005; Bonaparte & Sues, 2006; Soares, Martinelli & Oliveira, 2014; Romo de Vivar et al., 2020; Chambi-Trowell et al., 2021). Specimens of larger body sizes comprise a much smaller sample, and include mainly dinosaurs (*e.g.*, Bonaparte, Ferigolo & Ribeiro, 1999; Pretto et al., 2016; Müller, Langer & Dias-da Silva, 2018a) and dicynodonts (Araújo & Gonzaga, 1980). A purported pterosaur taxon, *Faxinalipterus minimus* Bonaparte, Schultz & Soares (2010), was named based on postcranial material (UFRGS-PV-0927-T), with a referred maxilla (UFRGS-PV-0769-T). All bones come from a single fossil site, known as Linha São Luiz (Faxinal do Soturno municipality, Rio Grande do Sul state), but were collected in two different field seasons (2002 and 2005) and come from two distinct sandstone blocks (Bonaparte, Schultz & Soares, 2010). Some authors have already argued against the referral of *Faxinalipterus minimus* to Pterosauria (*e.g.*, Soares et al., 2013; Dalla Vecchia, 2013), but none of them attempted an alternative attribution based on thorough comparisons with a broad sample of archosaurs. In addition, the attribution of the isolated maxilla to the taxon remained putative, and no further comments on this issue were made available in published literature. After further preparation efforts, the analysis of *Faxinalipterus minimus* holotype confirms that some elements have been misidentified. All bones referred to *Faxinalipterus minimus* are here redescribed, and the assignment of the maxilla (UFRGS-PV-0769-T) to the species is questioned. Finally, a new putative pterosauromorph is described based on a new specimen (CAPPA/UFSM 0300) from the *Faxinalipterus minimus* type locality.

## Geological Setting

**Figure 1:**
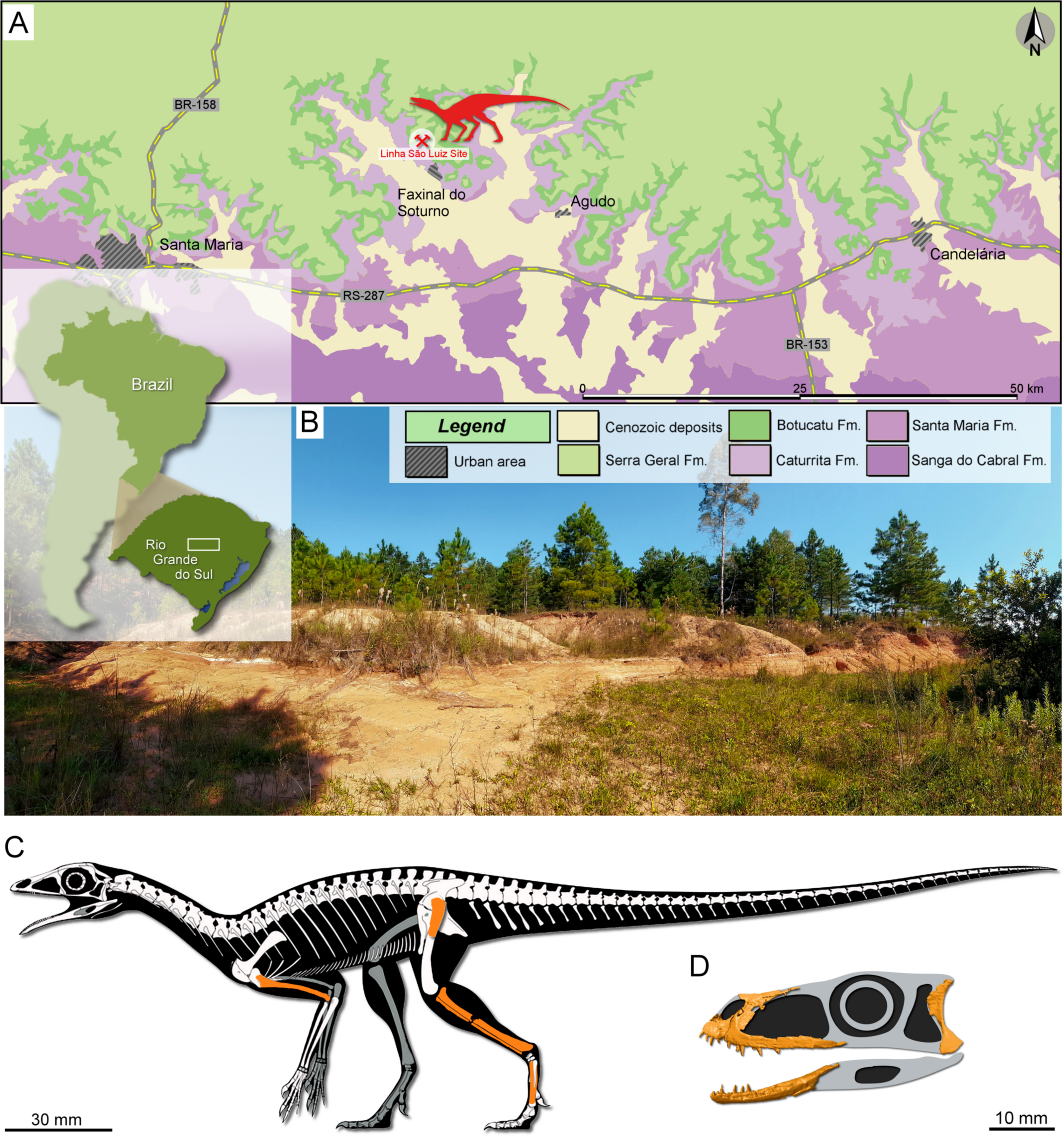
Study area and specimens. (A) Location map of the Linha São Luiz site and the surface distribution of the geologic units in the area. (B) General view of the Linha São Luiz site. (C) Hypothetical reconstruction of the skeleton (by Maurício S. Garcia) of *Faxinalipterus minimus* (UFRGS-PV-0927-T) depicting in orange the preserved elements (according to our reinterpretations). (D) Hypothetical reconstruction of the skull of *Maehary bonapartei* gen. et sp. nov. (CAPPA/UFSM 0300) depicting some of the preserved elements.

The sandy sedimentary package of the Paraná Basin in Rio Grande do Sul State, Southern Brazil, identified by *Andreis, Bossi & Montardo (1980)* as the Caturrita Formation, is nowadays interpreted as the top of a third-order continental sequence named Candelária Sequence (Horn et al., 2014) belonging to the Middle-Upper Triassic Santa Maria Supersequence (Zerfass et al., 2003). In this package is inserted the outcrop called Linha São Luiz (29°33′45″S; 53°26′48″W), located in the Faxinal do Soturno Municipality (Fig. 1). The Linha São Luiz site is about 20 m thick, being composed on its base of fine-grained and well-selected medium-grained sandstones with cross-bedded, low angle, stratification, followed by mostly fine-grained, well-sorted, massive sandstones with dispersed mud intraclasts. The middle portion of the exposition is composed of mudstones, and the upper portion is characterized by rhythmic sandstones and mudstones (Horn, Goldberg & Schultz, 2018). The specimens UFRGS-PV-0927-T (Fig. 1C), UFRGS-PV-0769-T, and CAPPA/UFSM 0300 (Fig. 1D), as well as most of the aforementioned recovered tetrapods, come from the massive sandstone facies (channel fill deposits). According to *Horn, Goldberg & Schultz (2018)*, this facies is the product of an ephemeral fluvial system generated by severe seasonal precipitation and catastrophic floods with high sediment load related to deconfinement or avulsion of hyperconcentrated flows. Recent dating by *Langer, Ramezani & Da-Rosa (2018)* based on zircon U-Pb analyses from massive sandstones pointed out an early Norian age (225.42 ± 0.37 Ma) for the Linha São Luiz site.

## Material and Methods

### Specimens

Regarding *Faxinalipterus minimus*, the specimen UFRGS-PV-0927-T is composed of some incomplete appendicular bones (Fig. 1C). According to Bonaparte, Schultz & Soares (2010: 64), it encompasses a left coracoid lacking the sternal end; the proximal portion of a left humerus; two fragments, possibly from a right humerus; proximal fragments of left radius and ulna; an almost complete left femur; an almost complete left tibia and fibula; fragments of right tibia and fibula associated with a possible metatarsal; and a few indeterminate fragments. All skeletal elements included in UFRGS-PV-0927-T were considered to belong to a single individual by those authors (“the material was found within a single small block of sandstone and considered to correspond to a single individual because of their size and structure”; p. 63). UFRGS-PV-0927-T was designated as the holotype of the purported pterosaur *Faxinalipterus minimus.* UFRGS-PV-0769-T was indicated as referred material and corresponds to a left maxilla preserving some teeth, found in another sand block. Unfortunately, there is no record of collection data regarding the stratigraphic position of the two specimens in the outcrop. Furthermore, as already commented, the specimen UFRGS-PV-0927-T was collected three years after the UFRGS-PV-0769-T, which makes it virtually impossible to associate them to the same individual. We consider that the skeletal elements of UFRGS-PV-0927-T plausibly belong to a single individual because of their close association in a small block, comparable size, common features (*i.e.*, hollowness and very thin cortex) and the absence of duplicated long bones. It should be noted that this specimen was on loan at the Museu Nacional/UFRJ that suffered a major fire destroying a great part of the collections (*e.g.*, Kellner, 2019). Fortunately, some elements of *Faxinalipterus*, including the maxilla UFRGS-PV-0769-T, were recovered. This is the second fossil vertebrate rescued from the Palace of the Quinta da Boa Vista Park to be studied (Kellner et al., 2019).

The specimen CAPPA/UFSM 0300 (Fig. 1D) comprises a small sandstone block with partially articulated cranial elements and some disarticulated postcranial bones (some vertebral centra and one scapula). The articulation degree, absence of duplicated bones, and similar sizes led us to identify CAPPA/UFSM 0300 as a single individual. Similarly, CAPPA/UFSM 300 was excavated several years after the discovery of *Faxinalipterus minimus* specimens. Hence, it is highly unlikely that it belongs to those previously known individuals.

### CT-scanning and three-dimensional reconstructions

UFRGS-PV-0769-T and CAPPA/UFSM 0300 were scanned using µCT scan Skyscan™ 1173. UFRGS-PV-0769-T was scanned at the Laboratório de Instrumentação Nuclear (LIN/COPPE), Universidade Federal do Rio de Janeiro (UFRJ), Rio de Janeiro (Brazil), using 130 kV and 61 µA, resulting in 2,240 tomographic slices, with a voxel size of 13 µm. CAPPA/UFSM 0300 was scanned at the Laboratório de Sedimentologia e Petrologia of the Pontifícia Universidade Católica do Rio Grande do Sul (PUCRS), Porto Alegre (Brazil), using 110 kV and 72 µA. The scan resulted in 1,748 tomographic slices, with a voxel size of 21.87 µm. Digital preparation and volume rendering were performed using Dragonfly2020.2 (Version 2020.2 [for Windows]. Object Research Systems (ORS), Inc., 2020). DesignSpark Mechanical (Version 2.0 for [for Windows]. Ansys, Inc., RS Components) was used to render the 3D models. The raw information of the CT-scans is available at MorphoSource (https://doi.org/10.17602/M2/M393025).

### Phylogenetic analysis

The holotype (UFRGS-PV-0927-T) of *Faxinalipterus minimus* and CAPPA/UFSM 0300 had their phylogenetic affinities investigated through their scores in Ezcurra et al. (2020) data matrix, which is a modified version of the data matrix originally published by *Ezcurra (2016)*. The final data matrix includes 823 characters and 196 operational taxonomic units (OTUs), but only 159 OTUs (including *Faxinalipterus minimus* and CAPPA/UFSM 0300) are active and one character is deactivated (following the former study). The data matrix was the subject of an equally weighted parsimony analysis in TNT v. 1.5 (Goloboff & Catalano, 2016). Following the approach by Ezcurra et al. (2020), 107 characters were treated as ordered (additive). *Petrolacosaurus kansensis* was used to root the most parsimonious trees (MPTs), which were recovered according the protocol reported by Ezcurra et al. (2020); *i.e.*, employing the new technology search algorithms until 100 optimal hits are reached, as in the former study. Then, topologies retained in overflowed replicates were branch-swapped for MPTs using TBR. The strict consensus tree was generated using all trees recovered in the analysis and all active OTUs. Decay indices (Bremer support values) were also obtained with TNT v. 1.5.

### Nomenclatural acts

The electronic version of this article in Portable Document Format (PDF) will represent a published work according to the International Commission on Zoological Nomenclature (ICZN), and hence the new names contained in the electronic version are effectively published under that Code from the electronic edition alone. This published work and the nomenclatural acts it contains have been registered in ZooBank, the online registration system for the ICZN. The ZooBank LSIDs (Life Science Identifiers) can be resolved, and the associated information viewed through any standard web browser by appending the LSID to the prefix http://zoobank.org/. The LSID for this publication is: urn:lsid:zoobank.org:pub:8C6EAD24-B978-45C3-A3BB-71D2F2E8E48F). The online version of this work is archived and available from the following digital repositories: PeerJ, PubMed Central and CLOCKSS.

## Results

### Systematic paleontology

**Table:** 

ARCHOSAUROMORPHA *von Huene, 1946* (Benton, 1985)
ARCHOSAURIFORMES *Gauthier, 1986* (Gauthier, Kluge & Rowe, 1988)
ARCHOSAURIA *Cope, 1869* (Gauthier & Padian, 2020)
PTEROSAUROMORPHA *Padian, 1997*
LAGERPETIDAE Arcucci, 1986 (*sensu*Nesbitt et al., 2009a; Nesbitt et al., 2009b)
Genus †*Faxinalipterus*Bonaparte, Schultz & Soares, 2010

**Amended diagnosis.** Same as for type and only species.

**Type and only species.** *Faxinalipterus minimus* Bonaparte, Schultz & Soares, 2010 (specific name amended; International Commission on Zoological Nomenclature: art. 34.2; ICZN, 1999).

**Holotype.** UFRGS-PV-0927-T: right humerus, two fragments of a left humerus, a possible proximal portion of a left femur, tibiae and fibulae, and two fragmentary metatarsals. All bones were associated within a single sandstone block and plausibly belong to a single individual.

**Locality and horizon.** Linha São Luiz Site (29°33′45″S; 53°26′448″W), deactivated quarry about 1.5 km northeast of the town of Faxinal do Soturno, Rio Grande do Sul State, Brazil; Santa Maria Supersequence, Upper portion of Candelária Sequence, early Norian (Soares, Schultz & Horn, 2011; Horn et al., 2014; Langer, Ramezani & Da-Rosa, 2018).

**Amended diagnosis.** *Faxinalipterus minimus* is a gracile and small archosaur that differs from all other known archosaurs based on a unique combination of character states: shafts of limb bones are hollow and thin-walled; gracile and elongated humerus with a triangular deltopectoral crest that extends down less than one-third of the total length of the bone; presence of a caudolateral longitudinal depression on the proximal portion of the humerus; poorly expanded femoral head; tibia with a poorly developed cnemial crest, a concave proximal articular surface, and lateral condyle offset cranially from the medial condyle; fibula with a rounded and reduced proximal articular surface, and a tubercle for the attachment of the iliofibularis muscle located on its proximal portion.

### Description and comparisons

The skeletal elements of *Faxinalipterus minimus* are sorted below according to our identification but also reporting the original identification by *Bonaparte, Schultz & Soares (2010)* and discussing it.

#### Forelimb

**Figure 2:**
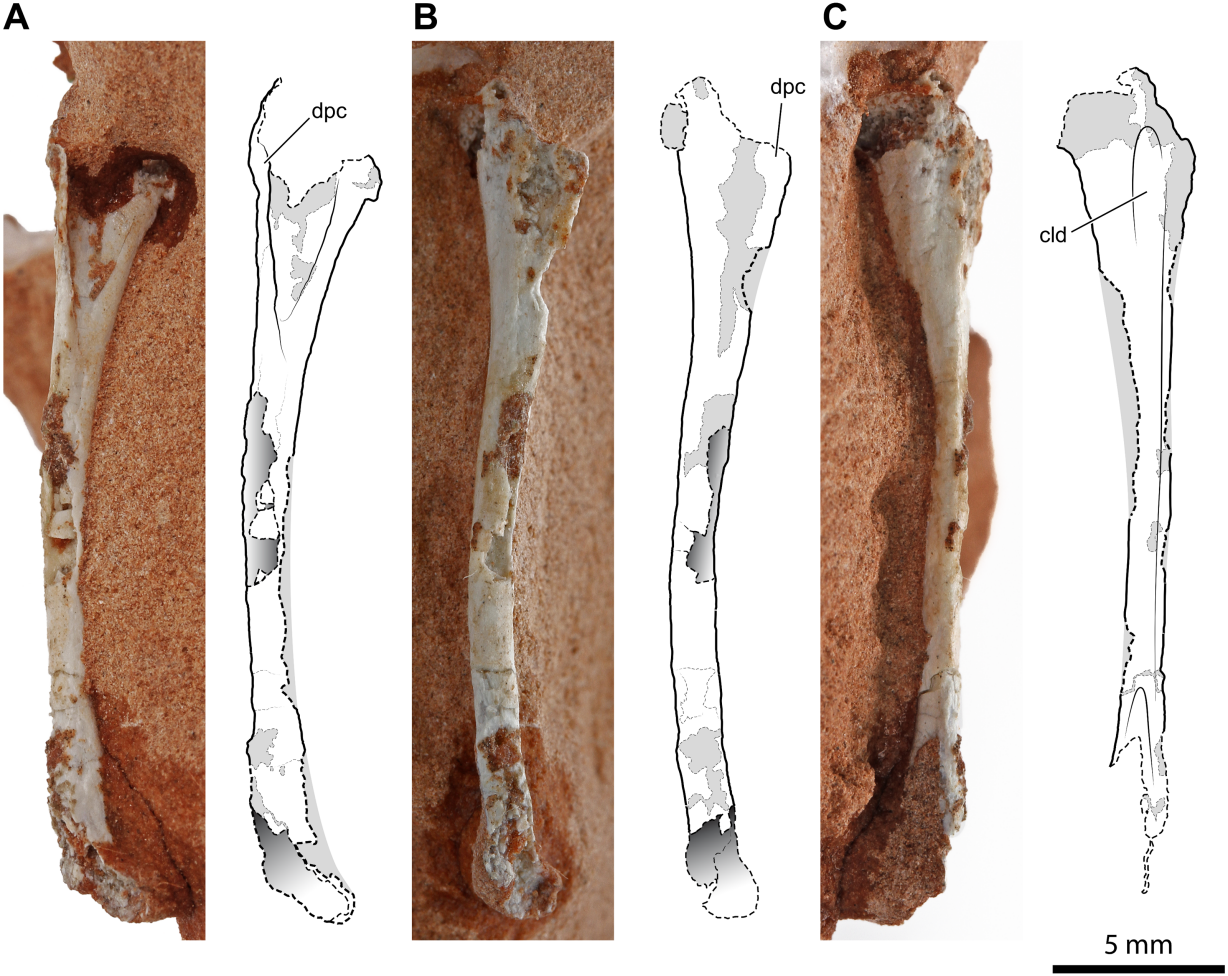
Right humerus of *Faxinalipterus minimus* (UFRGS-PV-0927-T). (A) Cranial view. (B) Lateral view. (C) Caudal view. cld, caudolateral depression; dpc, deltopectoral crest.

An almost complete right humerus (Fig. 2) is visible in cranial, medial, and caudal views on a sandstone fragment. Its total length is 25.8 mm; the craniocaudal, as well as the lateromedial diameter of the diaphysis, is 1.3 mm at mid-shaft (the cross-section is circular), but its proximal portion expands lateromedially to 4.3 mm. The element is long, slender and slightly bowed medially and cranially. The proximal articular head is continuous with the dorsal part of the deltopectoral crest; in caudal view, it is slightly set off medially to the axis of the diaphysis. The proximal expanded portion is deeply concave cranially. The deltopectoral crest is thin, triangular in lateral view (Fig. 2B), convex medially, and concave laterally. Its medial surface is damaged, but this does not alter the shape of the crest. The deltopectoral crest extends down the proximal end for up to about 28% of the inferred total length of the bone. In caudal view, the proximal expanded part of the humerus has a caudolateral longitudinal depression that is narrow and shallow (Fig. 2C), like in *Dibothrosuchus elaphros* (Simmons, 1965; Wu & Chatterjee, 1993). The shaft is slender and long. The damage at mid-shaft shows that the shaft is hollow inside and thin-walled. The distal end is slightly expanded, and the condyles are damaged.

*Bonaparte, Schultz & Soares (2010)* considered this bone as a pterosaur left femur. This misidentification was probably due to the partial preparation of the proximal part of the bone at the time of its description by these authors (see Bonaparte, Schultz & Soares, 2010: fig 4.1D). Actually, this bone is rather similar to the humeri of certain distinct Late Triassic archosaurs, as the lagerpetid *Lagerpeton chanarensis* Romer, 1971 (McCabe & Nesbitt, 2021) (early Carnian, Argentina) and the early-diverging crocodylomorphs *Terrestrisuchus gracilis* Crush, 1984 (Crush, 1984) (upper Norian or Rhaetian, UK) and *Hesperosuchus agilis* Colbert, 1952 (Colbert, 1952) (early Norian; Arizona, USA), in overall morphology and slenderness, shape of the expanded proximal portion (including the shape and position of the deltopectoral crest) and curvature of the shaft (Crush, 1984: figs. 7C–7F; Colbert, 1952: fig. 22). The ratio of the deltopectoral crest length to the total humerus length (about 0.28) is like that observed in most early crocodylomorphs, such as *Terrestrisuchus gracilis* (0.26; Crush, 1984), *Hesperosuchus agilis* (0.20; Colbert, 1952), *Sichuanosuchus shuhanensis* (0.23; Wu, Sues & Dong, 1997), and *Dibothrosuchus elaphros* (0.30; Wu & Chatterjee, 1993). Although larger than what is presumed for UFRGS-PV-0927-T, *Terrestrisuchus gracilis* and *Hesperosuchus agilis* were small animals (the humerus of *Terrestrisuchus gracilis* was about 45 mm long; Crush, 1984), and their bones are hollow and thin-walled.

**Figure 3:**
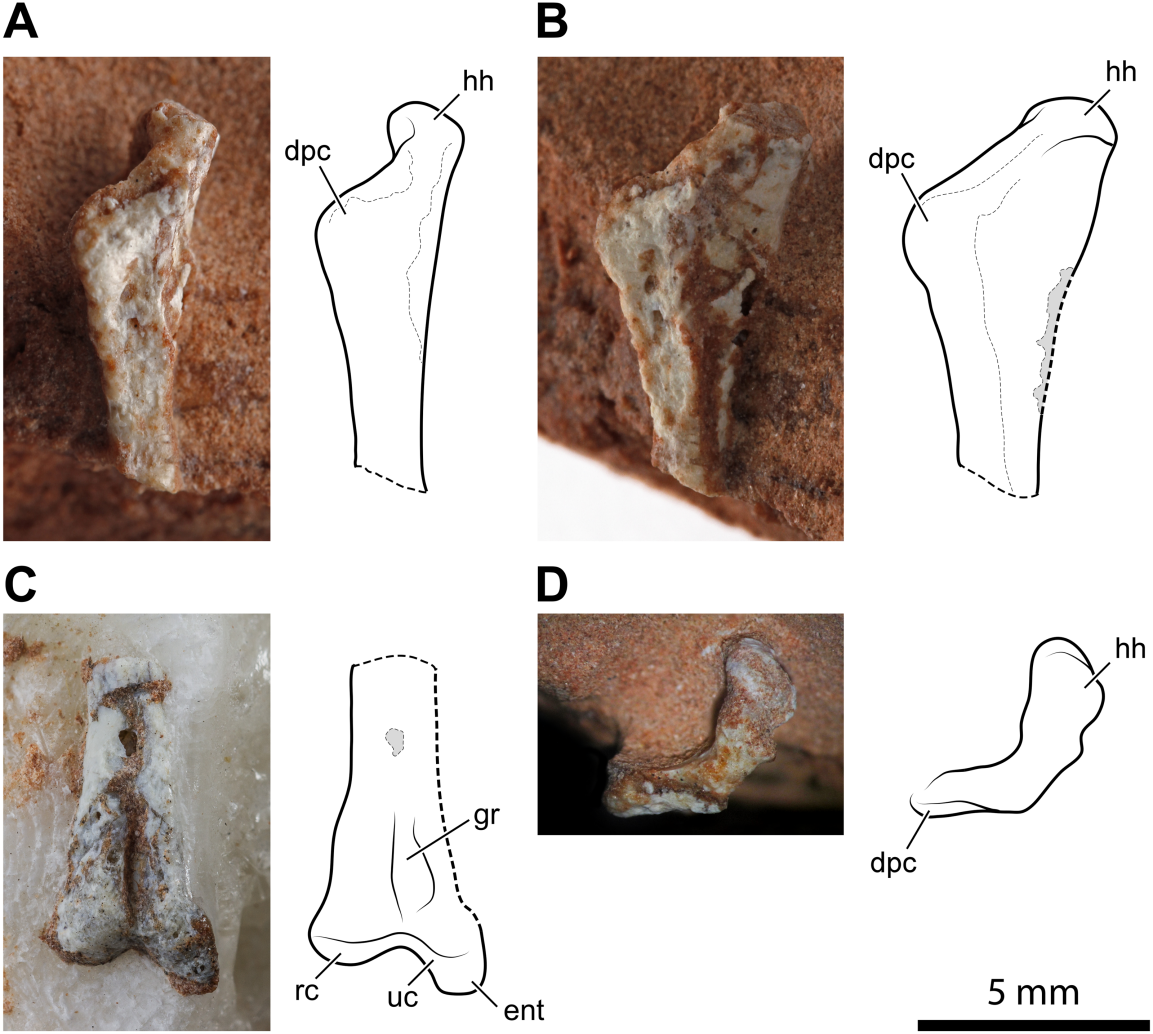
Left humerus of *Faxinalipterus minimus* (UFRGS-PV-0927-T). (A) Proximal portion in lateral view. (B) Proximal portion in caudolateral view. (C) Distal portion in caudal view. (D) Proximal portion in proximal view. dpc, deltopectoral crest; ent, entepicondyle; gr, groove; hh, humeral head; rc, radial condyle; uc, ulnar condyle.

Two fragments, preserved in two distinct small blocks of rock, can be referred to another humerus. If the bones of UFRGS-PV-0927-T belong to a single individual, as hypothesized by Bonaparte, Schultz & Soares (2010), this should be the left humerus. One of the two fragments is the proximal expanded portion of the humerus (Figs. 3A, 3B–3D) and is 8.6 mm in length as preserved. The fragment is damaged; the deltopectoral crest, as well as part of the caput, were mostly worn away. However, the cranial concavity is clearly identifiable and expanded 4.3 mm lateromedially. The cross-section of the proximal portion of the shaft is circular, and the diameter of the diaphysis is 1.5 mm. This fragment was identified as part of a pterosaur left coracoid by Bonaparte, Schultz & Soares (2010: figs. 4.1a and 4.2a). As noted by Dalla Vecchia (2014: 274), the coracoids of early pterosaurs are rather unlike the fragment from the Caturrita Formation, mostly having a flat and broad shaft (Jenkins Jr et al., 2001: fig. 2; Dalla Vecchia, 2014: figs. 4.1.40, 4.1.63, 4.1.99, 4.1.67), and not a rod-like shaft with a circular cross-section. None of the structures characterizing the pterosaur coracoid (fused scapula or a sutural surface for it, coracoid tubercle, biceps tubercle, and the lower tubercle bordering the glenoid; Bennett, 2003) can be identified in this fragment. Our tentative identification as the proximal portion of the left humerus is based on the comparison with the proximal part of the right humerus described above, allied to the fact that they are specular, and coincident in size, shape and thickness of the deltopectoral crest.

The second of the two fragments is the distal part of the humerus with the condyles for articulation with radius and ulna (Fig. 3C). As preserved, its length is 7.2 mm. Lateromedially, the diameter of the diaphysis is 1.5 mm at the proximal part of the fragment but, at the end of the condyles, it expands to 3.5 mm. The condyles are much better preserved than those of the right humerus. One condyle (the left in the exposed view) is sub-spherical, while the other has a tongue-like profile in the exposed view and projects further ventrally because of the entepicondyle. A similar condylar arrangement occurs in the lagerpetids *Lagerpeton chanarensis* (McCabe & Nesbitt, 2021), *Kongonaphon kely* (Kammerer et al., 2020), and *Ixalerpeton polesinensis* (Cabreira et al., 2016), but comparisons with the humeri of early crocodylomorphs as *Hesperosuchus agilis* (see Colbert, 1952: figs. 22b-c) and *Terrestrisuchus gracilis* (see Crush, 1984: figs. 7C–7D) also suggests a very close resemblance. The condyles are separated by a narrow, deep, and longitudinal groove that is emphasized by the crushing of the hollow and thin-walled shaft. A similar groove occurs on the caudal side of the humeri of *Hesperosuchus agilis* (see Colbert, 1952: fig. 22C) and *Terrestrisuchus gracilis* (see Crush, 1984: fig. 7D).

Bonaparte, Schultz & Soares (2010: fig. 4.1b and 4.2b) identified this fragment as a proximal portion of a pterosaur left humerus. Bonaparte, Schultz & Soares (2010) reported a saddle-shaped broader extremity in this bone, which was their main feature to refer UFRGS-PV-0927-T to Pterosauria. This mistaken identification was partly caused by the partial preparation of the fragment at the time of its description by these authors. The absence of a deltopectoral crest was supposed to be a result of its small size, purported ventral displacement of this structure, and rock covering (Bonaparte, Schultz & Soares, 2010). The complete preparation of this fragment has shown that there is no deltopectoral crest and no saddle-shape proximal articular surface. The absence of these structures indicates that this fragment is not the proximal part of a pterosaur humerus. The comparison with the skeletal elements of other archosaurs, as a consequence of the identification of the nearly complete right humerus, agrees with our new identification of this element.

#### Hind limb

**Figure 4:**
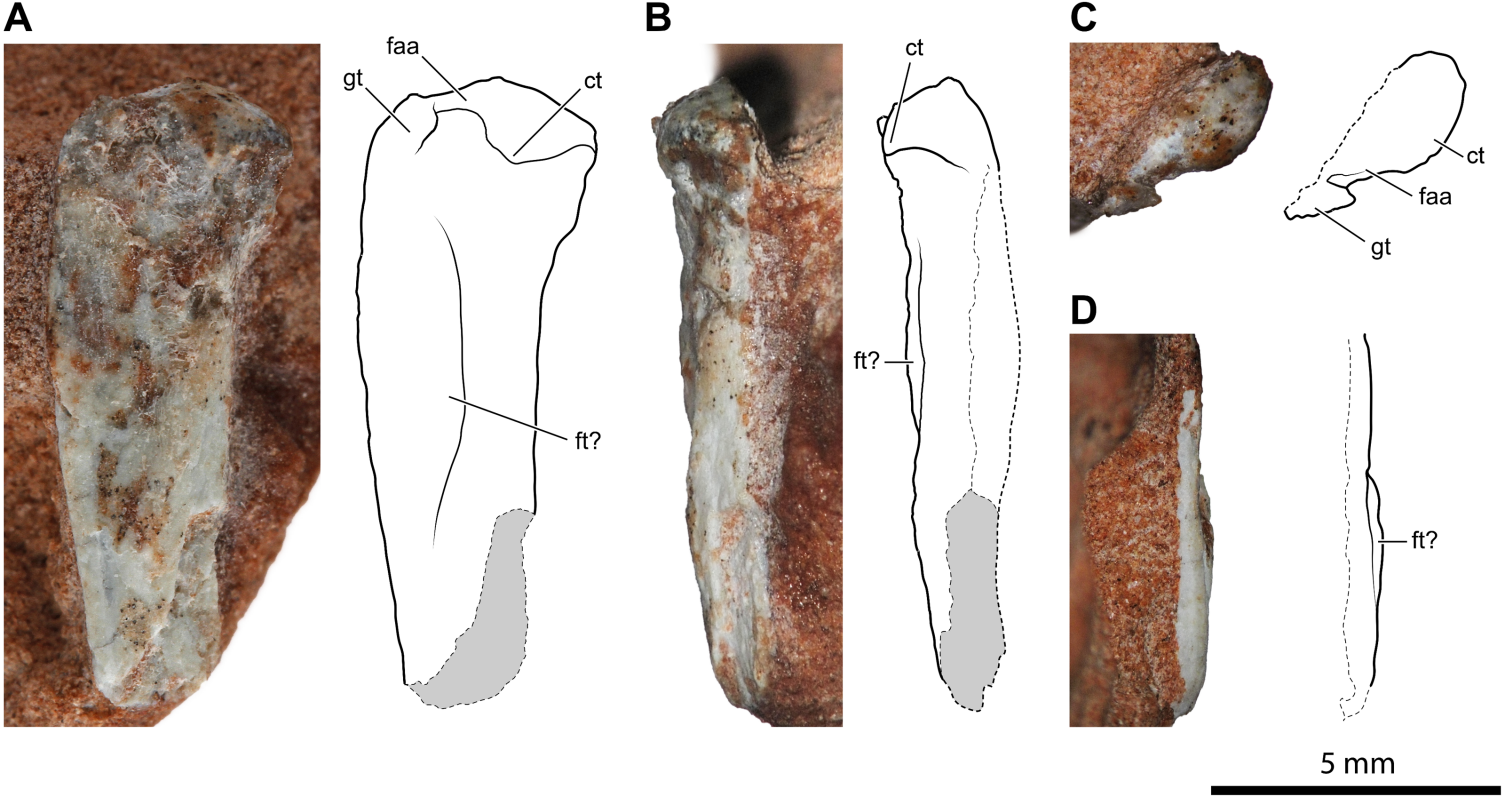
Proximal portion of a possible left femur of *Faxinalipterus minimus* (UFRGS-PV-0927-T). (A) Caudal view. (B) Medial view. (C) Proximal view. (D) Lateral view. ct, caudal tuber; faa, facies articularis antitrochanterica; ft, fourth trochanter; gt, greater trochanter.

A 14.2 mm-long fragment of a slender limb bone (Fig. 4) is tentatively identified as the proximal part of a left femur. The proximal epiphysis is moderately expanded mediolaterally. Nevertheless, it is less expanded than the femoral head of dinosaurs. There is no evidence of any groove on the proximal articular surface (Fig. 4C). The element lacks the cranial and the caudomedial tubers (*sensu* Ezcurra et al., 2020). Conversely, the caudal tuber is rounded and well-developed. This configuration of tubers is usually observed in lagerpetids (Nesbitt et al., 2009a; Cabreira et al., 2016; Ezcurra et al., 2020; Kammerer et al., 2020). On the other hand, the cranial and the caudomedial tubers are typically present in dinosauromorphs (Nesbitt, 2011; Ezcurra et al., 2020). Caudally to the caudal tuber, there is a concavity that corresponds to the trochanteric fossa (*sensu* Novas, 1996). This feature is regarded as an ornithodiran condition, occurring in some pterosaurs, lagerpetids, silesaurids, and dinosaurs (Novas, 1996; Nesbitt et al., 2010; Nesbitt, 2011; Ezcurra et al., 2020). The morphology of the transition between the femoral head and the shaft is obscured by the poorly preserved cranial portion of the bone. Actually, the preserved caudal portion of the transition resembles the condition observed in silesaurids (Nesbitt et al., 2010) and aphanosaurs (Nesbitt et al., 2017), where occurs a notch; however, the inaccessible cranial surface of the bone makes this assumption ambiguous. The proximal portion of the shaft has a low ridge running along the caudal margin of the head, which can be interpreted as a weakly developed greater trochanter (Fig. 4A). An arched longitudinal low ridge along the proximal portion of the shaft is identified as the fourth trochanter (Figs. 4A, 4B–4D). Both extremities of the crest merge smoothly with the shaft, resulting in a symmetrical profile. The broken shaft reveals that this possible femur is thin-walled and hollow inside, like the humeri described above. This bone was considered as an indeterminate fragment by Bonaparte, Schultz & Soares (2010: p. 64).

**Figure 5:**
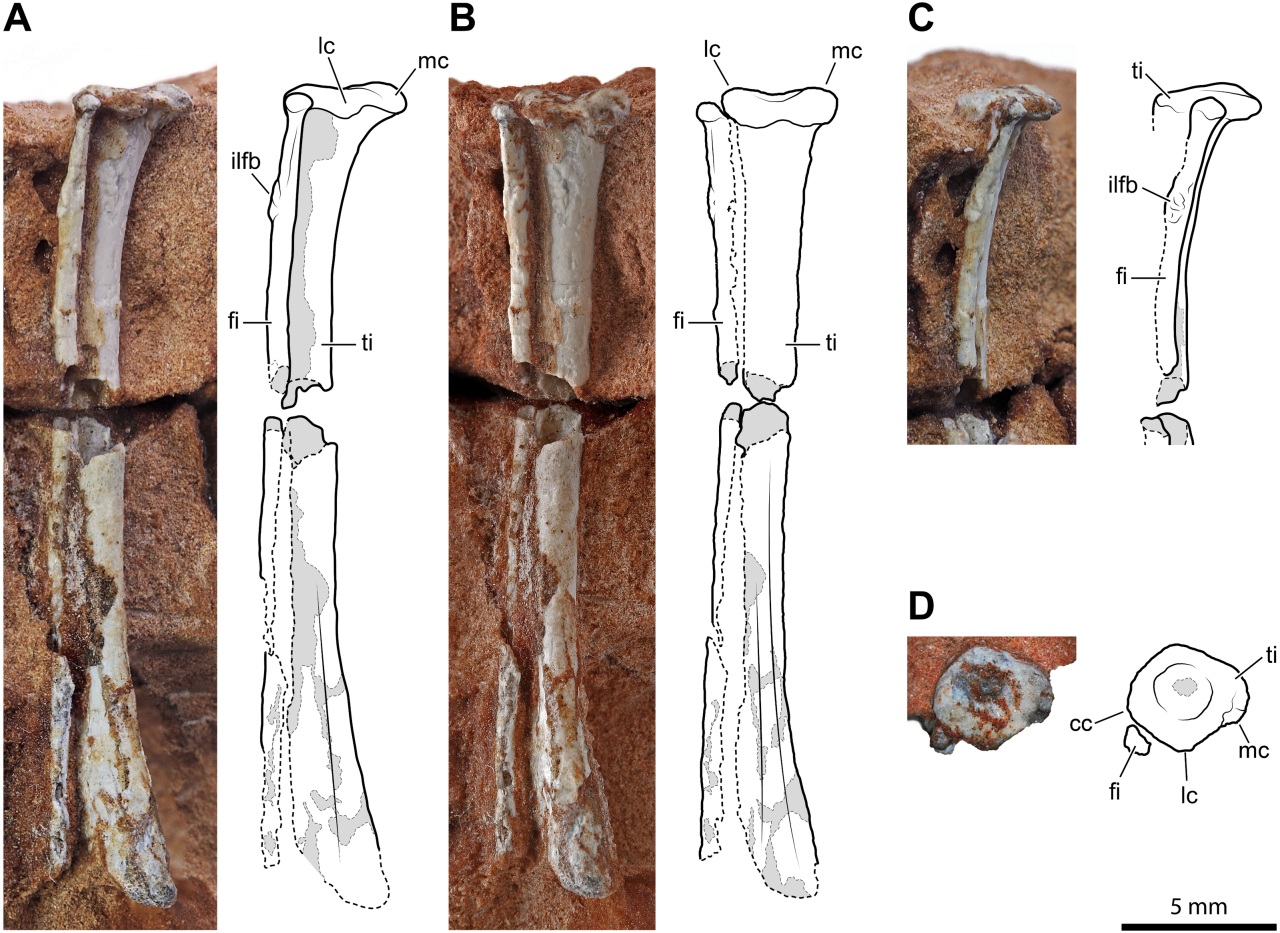
Left tibia and fibula of *Faxinalipterus minimus* (UFRGS-PV-0927-T). (A) Lateral view. (B) Caudal view. (C) Proximal portion in cranial view. (D) Proximal view. cc, cnemial crest; fi, fibula; ilfb, m. iliofibularis insertion; lc, lateral condyle; mc, medial condyle; ti, tibia.

An almost complete left tibia-fibula pair (Fig. 5) exposed in caudal view is preserved in two fragments of rock. The largest fragment fits the distal part and most of the diaphysis. As preserved, the tibia is >26.1 mm, and the fibula is >24.9 mm long, respectively, but the fibula is broken distally. They are not fused, neither proximally nor distally. They are both slender and elongated bones with a straight diaphysis, but the diameter nearly at the mid-shaft of the fibula is only 47% (0.75 mm) of that of the tibia. The proximal extremity of the tibia is expanded and nail head-shaped. The proximal articular surface is subcircular and flat, but with a central and circular depression (Fig. 5D). The diaphysis slightly tapers up to midway; then it expands again gradually and moderately. The expanded distal part is slightly bent medially. The transverse section of the bone is subcircular along most of its length, except on its distal portion, where it is somewhat flattened. The distal epiphysis does not bear distinct condyles, but this could be due to weathering. The damage at mid-shaft shows that the shaft is hollow inside and thin-walled (cortex is approximately 0.22 mm thick for a diameter of 1.6 mm).

The proximal extremity of the fibula is bulb-like, but it is not much expanded with respect to the diaphysis (Figs. 5A–5C). The latter maintains its diameter along the shaft and does not expand distally, even though it lacks its distal end. There is a tubercle for the attachment of the iliofibularis muscle on the proximal portion of the shaft. The damage at mid-shaft shows that its shaft is hollow inside and thin-walled (cortex is approximately 0.15 mm thick for a diameter of 0.75 mm).

Bonaparte, Schultz & Soares (2010: figs. 4.1e and 4.2e) identified these elements as a left tibia and fibula, which is followed here. On the other hand, the authors did not give any support to the attribution of those elements to Pterosauria, because their referral is based on the morphology of the proximal humerus (actually, the distal part of the humerus) and of the coracoid (much probably part of the other humerus). Like the bones under examination, the tibia is a straight elongated bone, and the fibula is much thinner than the tibia in non-pterodactyloid pterosaurs. However, tibia and fibula are usually coossified proximally in Triassic pterosaurs (Wellnhofer, 2003; Dalla Vecchia, 2013; Dalla Vecchia, 2014; Dalla Vecchia, 2021). In immature individuals, they are sutured with visible suture lines, but never separate as they are in the Brazilian remains. Also, the proximal termination of the tibia is never nailhead-like and proximally concave in pterosaurs (Dalla Vecchia, 2014: figs. 4.1.2, 4.1.45, 4.1.60, 4.1.95, 4.1.96, 4.1.126-127, and 4.1.171). The fibula tapers distally, ending before reaching the level of the tarsus in most non-pterodactyloid pterosaurs (Wellnhofer, 1978; Dalla Vecchia, 2003). In the exceptions *Austriadraco dallavecchiai* (Kellner, 2015) (Wellnhofer, 2003: fig. 15B), *Peteinosaurus zambellii* Wild, 1979 (Dalla Vecchia, 2003: fig. 3d-e), and *Campylognathoides liasicus* Quenstedt, 1858; (Wellnhofer, 1974: fig. 10), the fibula slightly expands distally and is never regularly cylindrical like in *F. minimus*. A wide spatium interosseum exists between tibia and fibula in the proximal third of the crus, which closes distally where the distal segment of the fibula is fused to the tibia (Dalla Vecchia, 2014: figs. 4.1.2, 4.1.45, 4.1.60, 4.1.95, 4.1.96, 4.1.126-127, and 4.1.171). Pterosaur fibulae are thicker at the midpoint of the spatium interosseum (*e.g.*, Wellnhofer, 2003: fig. 15B; Dalla Vecchia, 2014: figs. 4.1.45, 68, 126 and 171).

A sandstone fragment contains the incomplete remains (7.5 mm long) of two paired bones whose proportions are like those of the tibia-fibula described above. The slenderer, rod-like and cylindrical elements nearly reach the extremity of other, larger, bone. The terminal portion of the larger bone is expanded transversely, similarly to the proximal extremity of the tibia described above; however, its end is not flat but bears two sub-spherical condyles separated by a narrow intercondylar groove and projecting cranially. Before complete preparation, this fragment was identified as the proximal portion of a pterosaur left radius-ulna (Bonaparte, Schultz & Soares, 2010: figs. 4.1c and 4.2c), the ulna having “a modest olecranon process similar to that of *Preondactylus*” (p. 66). Further preparation has shown that the “olecranon process” of the purported ulna was only apparent. Furthermore, the diameter of the radius is almost the same as that of the ulna in basal pterosaurs (Dalla Vecchia, 2014; figs. 4.1.5, 4.1.7, 4.1.12, 4.1.22, 4.1.60, 4.1. 64-65, 4.1.81, 4.1.91-92, 4.1.96, 4.1.113, 4.1.137, 4.1.165, and 4.1.169). The proportions, size and the relative position of this pair of bones is similar to the tibia-fibula described above. Supposing, in absence of evidence on the contrary, that UFRGS-PV-0927-T is composed of bones from a single individual, the elements are here identified as the proximal part of the right tibia-fibula. The cnemial crest is poorly developed, as in the left tibia (Fig. 5D). This condition resembles that of pterosaurs (Ezcurra et al., 2020) and aphanosaurs (Nesbitt et al., 2017), as well as several pseudosuchians (*e.g.*, aetosaurs, loricatans, poposauroids). The lateral condyle is offset cranially from the medial condyle. This condition occurs in dinosauromorphs, lagerpetids, and proterosuchians (Langer & Benton, 2006; Nesbitt, 2011; Ezcurra et al., 2020). The tubercle for the attachment of the iliofibularis muscle lies on the proximal portion of the fibula (Fig. 5B), following the morphology of the left opposite element.

**Figure 6:**
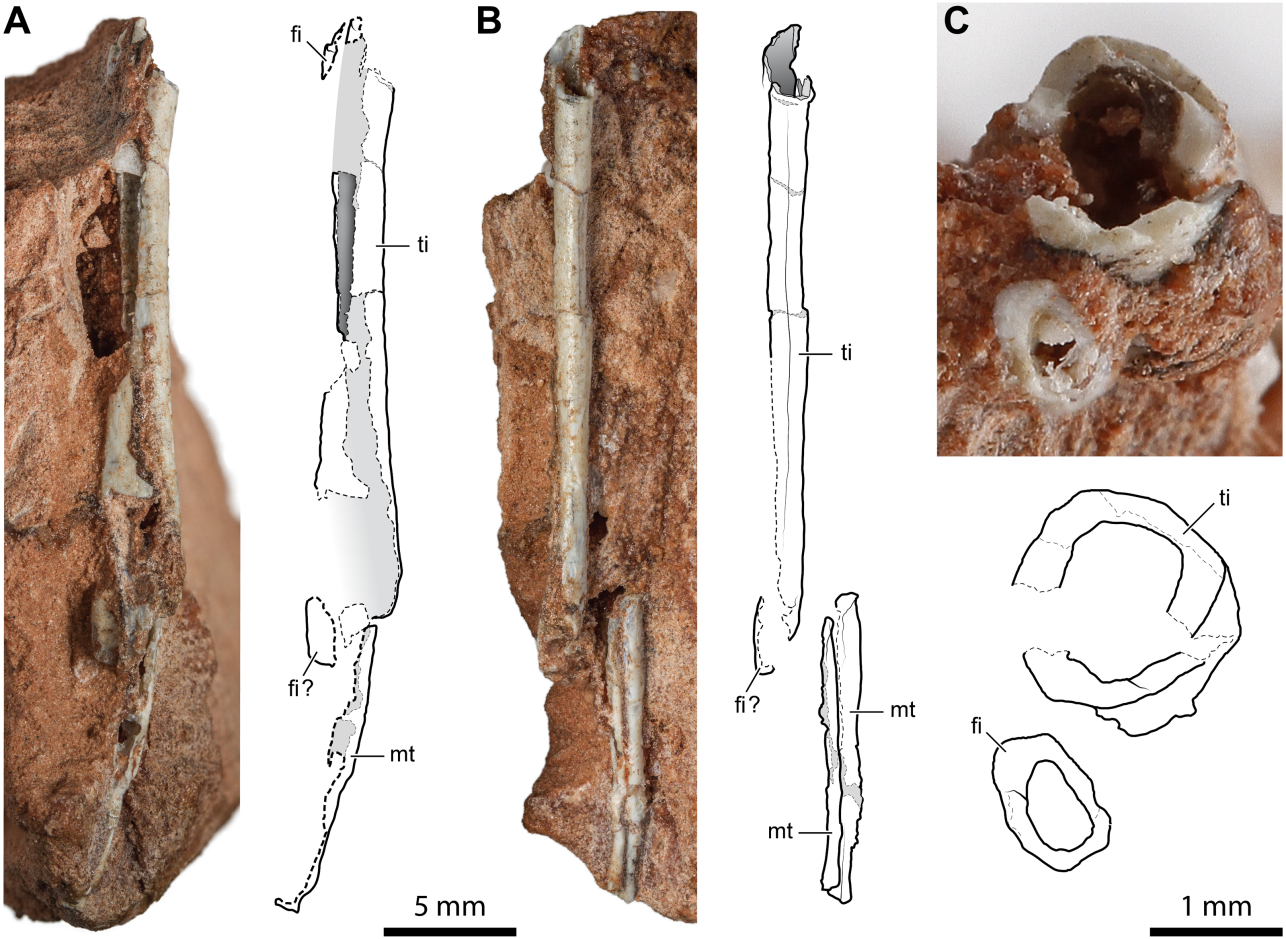
Diaphysis of the right tibia and some indeterminate metatarsals of *Faxinalipterus minimus* (UFRGS-PV-0927-T). (A) Caudal view. (B) Medial view. (C) Cross-section. fi, fibula; mt, metatarsal; ti, tibia.

The incomplete diaphyses of two paired long bones, with the aspect and proportions of the tibia-fibula pairs described above, are preserved in a rock fragment along with a couple of metapodials (Fig. 6). The more robust of the two paired bones has a straight tubular shaft with a hollow inside and a thin cortex (cortex thickness at the proximal extremity is ∼0.14 mm; Fig. 6C). Its fragmentary diaphysis is sub-circular in cross-section and is 25.2 mm long. The other bone is not exposed, but it can be seen in a cross-section close to the presumed proximal end of the more robust bone. It extends for 19.2 mm beneath and parallel to the diaphysis of the other bone. The elements interpreted as metapodials consist of two elongated, straight, and tubular elements that are parallel and closely set (Fig. 6B). They are 12.2 mm and 10.6 mm long, respectively.

*Bonaparte, Schultz & Soares (2010)* tentatively identified these bones as fragmentary right tibia and fibula (although only the tibia is shown in figs. 4.1F and 4.2F) associated with two indeterminate metatarsals. The identification of *Bonaparte, Schultz & Soares (2010)* is followed here. Nevertheless, these elements are too fragmentary, lacking the epiphyses or additional diagnostic traits. Therefore, the identification is mainly based on the proportions between the putative tibia and fibula.

### Systematic Paleontology

**Table:** 

ARCHOSAURIFORMES *Gauthier, 1986* (Gauthier, Kluge & Rowe, 1988)
ARCHOSAURIA *Cope, 1869* (Gauthier & Padian, 2020)
ORNITHODIRA *Gauthier, 1986* (PAN-AVES [Bibr ref-44][Bibr ref-35])
Genus †*Maehary* gen. nov.

**Generic etymology.** After Ma’ehary, a Guarani-Kaiowa locution that roughly means “who looks to the sky”. This alludes to the putative pterosauromorph affinities of the taxon.

**Generic diagnosis.** Same as for type and only species.

**Type and only species.** *Maehary bonapartei* sp. nov.

**Specific etymology.** In honor of Dr. José F. Bonaparte, a prominent Argentine paleontologist, for his tremendous contribution to the development of vertebrate paleontology in South America.

**Figure 7:**
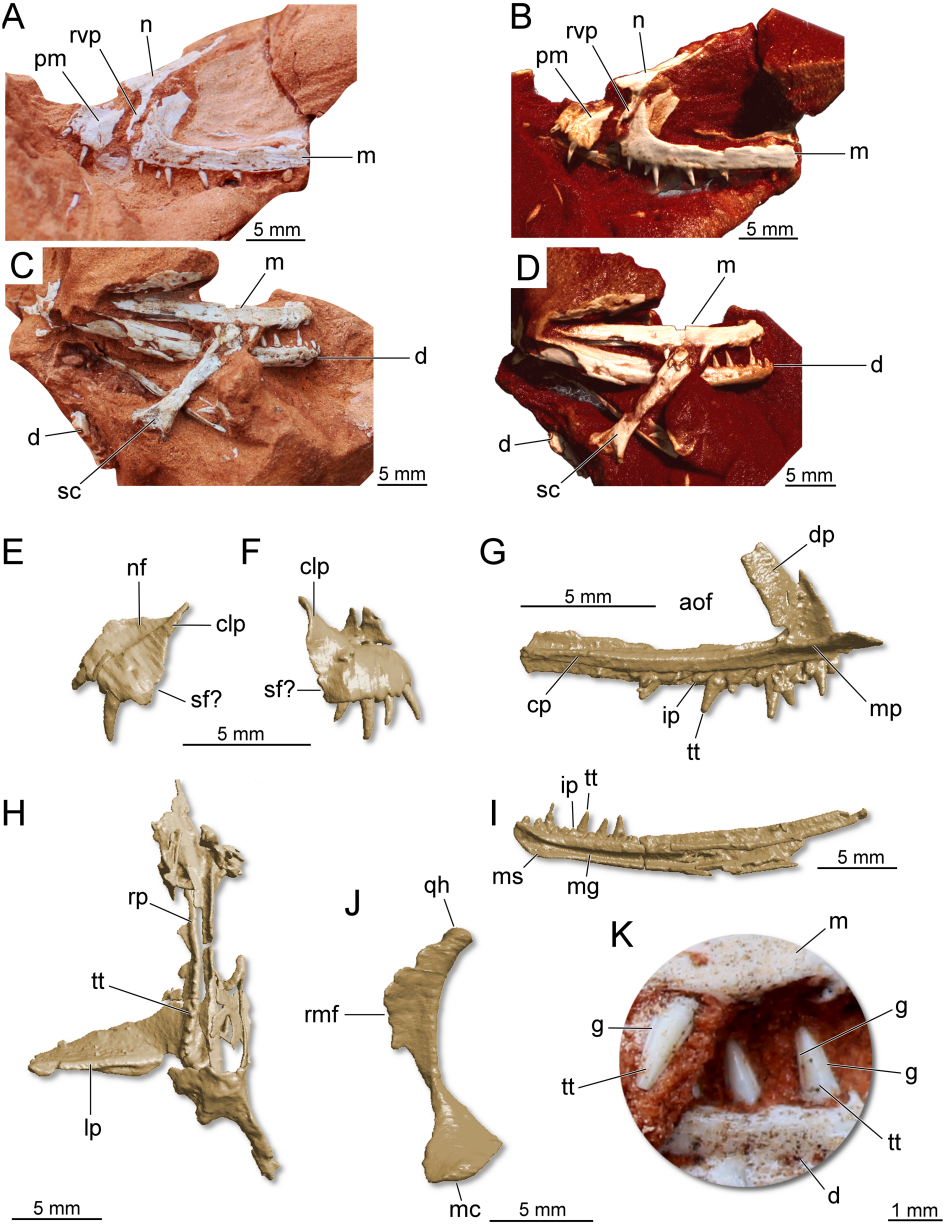
Partial skull of *Maehary bonapartei* gen. et sp. nov. (CAPPA/UFSM 0300). (A) Left lateral view. (B) Three-dimensional rendering in left lateral view. (C) Right lateral view. (D) Three-dimensional rendering in right lateral view. (E) Three-dimensional rendering of the left premaxilla in lateral view. (F) Three-dimensional rendering of the right premaxilla in lateral view. (G) Three-dimensional rendering of the left maxilla in medial view. (H) Three-dimensional rendering of the right pterygoid in ventral view. (I) Three-dimensional rendering of right dentary in medial view. (J) Three-dimensional rendering of the right quadrate in medial view. (K) Right maxillary and dentary teeth in labial view. aof, antorbital fenestra; cp, caudal process; clp, caudolateral process; d, dentary; dp, dorsal process; g, groove; ip, interdental plate; lp, lateral process; m, maxilla; mc, medial condyle; mg, Meckelian groove; mp, medial process; ms, mandibular symphysis; n, nasal; nf, narial fossa; pm premaxilla; rmf, rostromedial flange; qh, quadrate head; rp, rostral process; rvp, rostroventral process; sc, scapula; sf, subnarial foramen; tt, tooth.

**Holotype.** CAPPA/UFSM 0300 (Fig. 7), a partial skull, partial lower jaw, some vertebral centra, and a fragmentary scapula.

**Figure 8:**
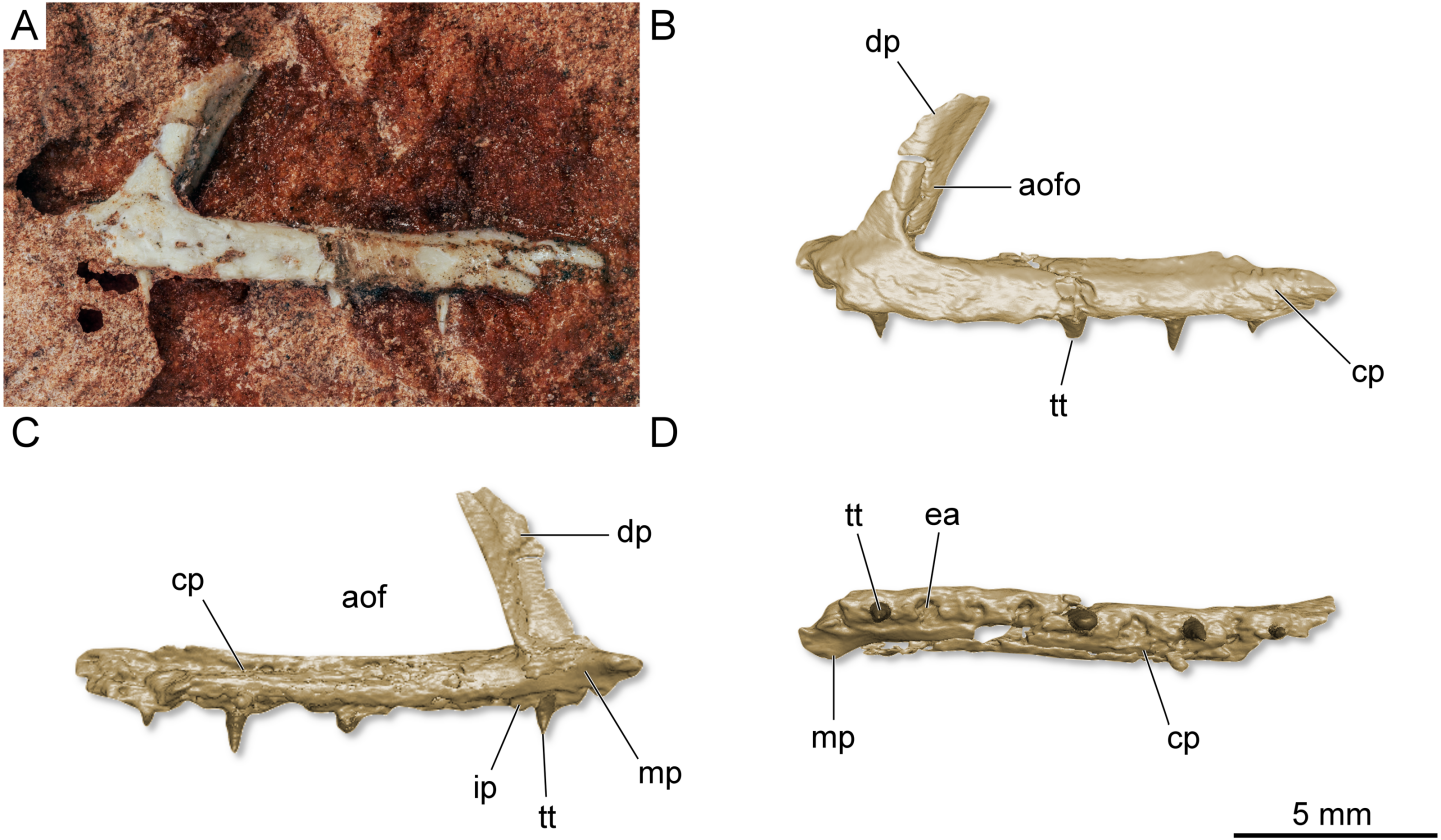
Left maxilla of *Maehary bonapartei* gen. et sp. nov. (UFRGS-PV-0769-T). (A) Lateral view. (B) Three-dimensional rendering in lateral view. (C) Three-dimensional rendering medial view. (D) Three-dimensional rendering in ventral view. aof, antorbital fenestra; aofo, antorbital fossa; cp, caudal process; dp, dorsal process; ea, empty alveolus; ip, interdental plate; mp, medial process; tt, tooth.

**Referred specimen.** UFRGS-PV-0769-T (Fig. 8), a left maxilla previously referred to *Faxinalipterus minimus* (Bonaparte, Schultz & Soares, 2010).

**Locality and horizon.** The holotype and referred specimen were excavated from the Linha São Luiz Site (29° 33 ′45″ S; 53° 26 ′48″ W), about 1.5 km northeast of the town of Faxinal do Soturno, Rio Grande do Sul State, Brazil; Santa Maria Supersequence, Candelária Sequence, early Norian (Horn et al., 2014; Soares, Schultz & Horn, 2011; Langer, Ramezani & Da-Rosa, 2018).

**Specific diagnosis.** *Maehary bonapartei* differs from all other known archosaurs based on a unique combination of character states (*autapomorphy): premaxilla with a deep narial fossa bounded caudoventrally by a sharp margin; slender maxilla with a wide antorbital fenestra (*i.e.*, dorsal process is rostrocaudally narrow) and an antorbital fossa restricted to its dorsal process; elongated rostroventral process of the nasal; lateral margin of the nasal is poorly developed, not forming a lateral shelf; strongly arched quadrate; slender dentary with a rounded rostral end; maxillary and dentary teeth without serrations and with two apicobasally oriented grooves*; and presence of palatal teeth on the rostral process of the pterygoid.

### Description and comparisons

The following description is preliminary and mainly focused on the holotype CAPPA/UFSM 0300. A comprehensive anatomical description of this taxon will be published posteriorly. The premaxilla is roughly trapezoidal in lateral view (Figs. 7E–7F). There is a deep narial fossa, which is caudally bordered by a ridge that extends along the caudolateral process. The caudal margin of the premaxilla bears a faint concavity, which may correspond to the rostral margin of the subnarial foramen. The maxilla is slender and triradiate, where the caudal (horizontal) process is the longest (Figs. 7A–7G). The rostral process is short, whereas in early crocodylomorphs (*e.g.*, *Dromicosuchus grallator* and *Sphenosuchus acutus*) it is well-developed rostrally (Irmis, Nesbitt & Sues, 2015). The dorsal (ascending) process is rostrocaudally short and slightly caudally oriented. Its distal tip is not preserved. Distinct from theropods (Martinez et al., 2011) and some sauropodomorph dinosaurs (Müller, Langer & Dias-da Silva, 2018a), the maxilla lacks a promaxillary fenestra. The antorbital fossa excavates the caudal half of the dorsal process, but otherwise is poorly developed. In lateral view, the fossa is not visible along the caudal process, as in the lagerpetid *Kongonaphon kely* (Kammerer et al., 2020). The dorsal and ventral margins of the caudal process run parallel along its horizontal axis, tapering at the distal end. Whereas some foramina occur on the lateral surface of the caudal process, a longitudinal ridge is absent. Nevertheless, the dorsolateral margin of the caudal process is bordered by a sharp ridge. The morphology of the caudal process of the maxilla indicates the presence of an enlarged antorbital fenestra. A similar condition occurs in gracilisuchids (*e.g.*, *Turfanosuchus dabanensis*; *Gracilisuchus stipanicicorum*; Butler et al., 2014) and aphanosaurs (*e.g.*, *Teleocrater rhadinus*; Nesbitt et al., 2017). The nasal is elongated and transversely narrow. The dorsal surface of the nasal is slightly deflected, resembling the “roman nose” of early loricatans and some sauropodomorphs (Nesbitt, 2011; Sereno, Martínez & Alcober, 2013; Müller, Langer & Dias-da Silva, 2018a). It lacks well-developed lateral shelves, which usually roof the antorbital fenestra in gracilisuchid pseudosuchians (Butler et al., 2014) and early sauropodomorph dinosaurs (Sereno, Martínez & Alcober, 2013). The rostroventral process is elongated and rests on the entire rostral margin of the dorsal process of the maxilla (Figs. 7A–7B). A well-developed rostroventral process is shared with gracilisuchids (Butler et al., 2014). The rostral surface of the rostroventral process receives the caudal surface of the premaxilla. The quadrate is strongly arched, with a caudal concave surface (Fig. 7J).

The hemimandibles are slender (one mm in height) and elongated (23 mm in length) (Fig. 7I). The dorsal and ventral margins of the dentary run parallel along the longitudinal axis, and the dentary does not taper rostrally, having a blunt rostral tip. This condition differs from silesaurids, lagerpetids, some pterosaurs, and some aetosaurs (Nesbitt et al., 2010; Nesbitt, 2011; Ezcurra et al., 2020). Furthermore, the rostral tip of the dentary is not ventrally or dorsally deflected and lacks a ventrally expanded process. Conversely, the long axis of the dentary curves upwards, a feature which is widespread in pseudosuchians. The density of foramina at the rostral end of the jaws resembles early dinosaurs (Sereno, Martínez & Alcober, 2013; Cabreira et al., 2016; Müller et al., 2018b). There is a longitudinal sulcus on the lateral surface of the bone, which houses a row of foramina (Fig. 7D). The alveolar line is slightly concave in lateral view; however, the caudal portion of the dentary lacks a dorsal expansion (= coronoid process). The mandibular symphysis is limited to the rostralmost tip of the dentary, differing from the longer symphysis of ornithosuchids (Nesbitt, 2011; Von Baczko & Ezcurra, 2013; Müller et al., 2020). Caudal to the symphysis, there is a wide Meckelian groove, which almost reaches the ventral margin of the element, as in *Ixalerpeton polesinensis* (Ezcurra et al., 2020). Triangular interdental plates are visible in medial view, differing from lagerpetids and pterosaurs, which lack interdental plates (Ezcurra et al., 2020). The mandibular fenestra is not entirely preserved. Therefore, its dimensions and shape are uncertain.

There are four premaxillary teeth (Fig. 7F), a widely distributed condition among early archosaurs (Nesbitt, 2011; Ezcurra et al., 2020). This condition differs from the saurischians *Tawa hallae* (Nesbitt et al., 2009a; Nesbitt et al., 2009b), *Gnathovorax cabreirai* (Pacheco et al., 2019), and *Daemonosaurus chauliodus* (Nesbitt et al., 2021), which bear three teeth in each premaxilla. The premaxillary teeth are cylindrical, pointed, and distally recurved. No serrations, cuspids, or sulci are present in the premaxillary teeth. The fourth tooth is the smallest in height. The left maxilla preserves 11 tooth positions. Six are occupied by teeth that resemble the premaxillary ones in shape, albeit being almost straight along their apicobasal axis. The rostralmost tooth is the smallest, whereas the third tooth is the largest among the preserved elements. The teeth lack serrations (Fig. 7K), a peculiar condition among archosaurs that occurs in pterosauromorphs (*i.e.*, lagerpetids and pterosaurs; sensu (Ezcurra et al., 2020)). Another peculiar feature in the maxillary teeth is the presence of two apicobasal sulci running on the mesial and distal margins of the labial surface (Fig. 7K), in a condition that seems to be unique. This feature is repeated in the lower jaw teeth. The dentary bear about 13 preserved tooth positions. The first seven alveoli are filled with complete or partially preserved teeth. The dentary teeth are cylindrical and pointed, such as the upper jaw teeth. There are no diastemas between the elements and no empty space between the rostral end of the bone and the first tooth. The latter condition differs from early sauropodomorph dinosaurs (Cabreira et al., 2016). The first tooth is partially preserved, being slightly distally inclined. The three last teeth are the only entirely preserved. These are almost equal in size. The specimen preserves eight palatal teeth on the rostral (palatal) process of the pterygoid (Fig. 7H). These tiny teeth are aligned parasagittally and featureless, such as in the early dinosauriform *Lewisuchus admixtus* (Bittencourt et al., 2015) and early dinosaurs (Martinez et al., 2011; Sereno, Martínez & Alcober, 2013; Müller et al., 2018b). Unlike *Eoraptor lunensis* (Sereno, Martínez & Alcober, 2013), there are no teeth on the lateral ramus of the pterygoid.

### Phylogenetic analysis

The phylogenetic analysis recovered 560 most parsimonious trees (MPTs) of 5,016 steps each, with a consistency index of 0.214 and a retention index of 0.668. In all the MPTs, the holotype of *Faxinalipterus minimus* nests in a polytomy within Lagerpetidae. These affinities are supported by: (i) an enlarged caudal tuber on the proximal portion of the femur (497: 0 →1); and (ii) absence of a cranial tuber on the femoral head (498: 0 →1). Conversely, if femoral characters are treated as missing entries, *Faxinalipterus minimus* nests in a large polytomy in the base of Archosauria. Therefore, an assignation to Lagerpetidae relies solely on the femoral traits. *Maehary bonapartei* nests as the earliest-diverging member of Pterosauromorpha (*sensu* Ezcurra et al., 2020; fig. 10) in all the MPTs. This position is supported by: (i) a thin caudonarial process of premaxilla (37: 0 →1), it is also found in saurischian dinosaurs and in non-poposauroid early suchians; (ii) a concave rostral margin of the dorsal process of maxilla (59: 0 →1), it is widespread in archosauriforms; and (iii) maxillary/dentary teeth without serrations (304: 2 →0). One additional step is necessary to recover *Faxinalipterus minimus* as the sister taxon to *Maehary bonapartei.* Affinities of the main clades follow the topology presented by Ezcurra et al. (2020), where Lagerpetidae is the sister-group to Pterosauria, both comprising less inclusive clades within Pterosauromorpha. The latter nests as the sister-group to Dinosauromorpha. Finally, aphanosaurs are the sister-group to Ornithodira, such as proposed by *Nesbitt et al. (2017)*. The inner affinities of Aphanosauria and Lagerpetidae are poorly resolved in the strict consensus tree.

## Discussion

### Faxinalipterus minimus

The assignment of *Faxinalipterus minimus* to Pterosauria by *Bonaparte, Schultz & Soares (2010)* was primarily based on the purported saddle-shaped morphology of the head of the ‘humerus’ (which is apomorphic for pterosaurs; Bennett, 1996; Kellner, 1996; Andres, 2010; Soares et al., 2013), the general morphology of the ‘coracoid’, and the hollow long bones with thin cortex. However, those bones are quite unlike the humeri and coracoids of Triassic pterosaurs. Further preparation revealed that the element originally referred as ‘proximal humerus’ is probably the distal portion of a humerus. The element identified by Bonaparte, Schultz & Soares (2010) as a femur is highly similar to the humeri of certain distinct archosaurs, as early-diverging crocodylomorphs and lagerpetids. Also, as discussed above, the element previously referred to as a ‘coracoid’ is most probably the proximal portion of the correlative left humerus.

Hollow long bones with a cortical thickness comparable or even thinner than that of UFRGS-PV-0927-T occur in: theropod dinosaurs (Colbert, 1989; Padian, Horner & de Ricqlès, 2004; Nesbitt, 2011); early crocodylomorphs (Colbert, 1952; Crush, 1984); the shuvosaurids *Effigia okeeffeae* and *Shuvosaurus inexpectatus* (see *Nesbitt, 2011)*; the lagerpetids *Dromomeron romeri* and *D. gregori* (see *Nesbitt, 2011)*; the silesaurids *Asilisaurus kongwe*, *Eucoelophysis baldwini* and *Silesaurus opolensis* (see *Nesbitt, 2011)*, the tanystropheid *Langobardisaurus pandolfi* (see *Saller, Renesto & Vecchia, 2013; Holgado et al., 2015)*; drepanosaurids (Renesto, 1994; Renesto et al., 2010); the sharovipterygids *Sharovipteryx mirabilis* (see Gans, Darevski & Tatarinov, 1987) and *Ozimek volans* (see Dzik & Sulej, 2016), and possibly also the kuehneosaurids (Colbert, 1966; Evans, 2009). Therefore, hollow and thin-walled bones only indicate that *Faxinalipterus minimus* was a lightly built sauropsid, and most probably belongs to one of the taxa listed above.

*Soares et al. (2013)* were the first to question the pterosaurian nature of *Faxinalipterus minimus* and pointed out some misidentification of the postcranial elements. They also indicated that the maxilla (UFRGS-PV-0769-T) was not directly associated with the holotype. Dalla Vecchia (2014: p. 274) argued against the assignment of *Faxinalipterus minimus* to Pterosauria by Bonaparte, Schultz & Soares (2010: 64), which does not report any apomorphic features but only a purportedly diagnostic combination of character states. “Fibula not fused to the tibia”, for instance, could be a consequence of early ontogeny if *Faxinalipterus minimus* was a pterosaur (*e.g.,* Kellner, 2015; Jiang et al., 2021). “Fibula... the same length as the tibia” is the plesiomorphic condition in tetrapods and occurs in some pterosaurs like *Campylognathoides* sp. (Wellnhofer, 1974) and *Austriadraco dallavecchiai* (see Wellnhofer, 2003; Kellner, 2015). “Fibula with a distal expansion” is a mistake because the fibula of UFRGS-PV-0927-T does not preserve their distal ends. Furthermore, it is unclear what *Bonaparte, Schultz & Soares (2010)* mean for “major tuberosity” of the humerus, because there is no distinct process in the presumed humerus other than its purported saddle-like articular head. Finally, there is nothing like an “acrocoracoid process” (= biceps tubercle) in the purported ‘coracoid’. Therefore, according to the new interpretations presented here, *Faxinalipterus minimus* (UFRGS-PV-0927-T) fails to show any feature exclusively shared with pterosaurs (*e.g.*, Fernandes, Nunes & Costa, 2021). The exclusion of *Faxinalipterus minimus* from Pterosauria makes this taxon part of an extensive list of Late Triassic species mistakenly referred to Pterosauria in their original descriptions (Peyer, 1931; Olsen, 1979; Olsen, 1980; Carroll, 1988, Fraser, 1988; Wellnhofer, 1991; Renesto & Fraser, 2003; Dzik et al., 2008; Renesto et al., 2010, Dalla Vecchia, 1994; Dalla Vecchia, 2013; Dalla Vecchia, 2014; Dalla Vecchia & Cau, 2015; Holgado et al., 2015).

Conversely, the affinities of this taxon remain somewhat obscure. Whereas the humeral morphology resembles that of early crocodylomorphs (*e.g.*, *Terrestrisuchus gracilis*) and lagerpetids (*e.g.*, *Lagerpeton chanarensis*), it lacks the caudal hook (= humeral hooked process), typical of crocodylomorphs (Colbert, 1952; Bonaparte, 1972; Crush, 1984; Nesbitt, 2011; Leardi, Yáñez & Pol, 2020). The possible femur bears a set of tubers that matches with the condition of lagerpetids; however, the femoral head is not hook-like, differing from the specimens referred to this clade. The non-hook-like morphology does not seem related to diagenetic biases. The tibia and fibula bear peculiar traits (see above) that are shared with both, pseudosuchians and ornithodirans. The holotype of *Faxinalipterus minimus* carries a unique combination of traits (see “Amended diagnosis”) that sustain its taxonomic validity. On the other hand, this challenging taxon displays several features shared by distinct Late Triassic archosaurs, hindering efforts to place it in a concrete lineage within Archosauria. So, a less inclusive classification demands the discovery of further specimens. For instance, some anatomical traits may suggest crocodylomorph affinities, such as the thin-walled bones (not restricted to crocodylomorphs) and the presence of a caudolateral depression on the proximal portion of the humerus. A similar caudolateral depression occurs in *Dibothrosuchus elaphros* (Simmons, 1965; Wu & Chatterjee, 1993). Basal crocodylomorphs have not been reported yet from the Caturrita Formation, but they were found in the late Carnian Ischigualasto Formation (*Trialestes romeri*) and the late Norian Los Colorados Formation (*Pseudhesperosuchus jachaleri*) of NW Argentina (Bonaparte, 1972; Irmis, Nesbitt & Sues, 2015; Lecuona, Ezcurra & Irmis, 2016). *Mattar (1987)* referred *Barbarenasuchus brasiliensis* from the Middle to Late Triassic Santa Maria Formation of Brazil to the Crocodylomorpha, but this referral is considered dubious (França, Bittencourt & Langer, 2013; Leardi, Yáñez & Pol, 2020). Therefore, on the assumption that *Faxinalipterus minimus* represents an early-diverging crocodylomorph, it would be the first record of the group from the Upper Triassic sediments of Brazil, expanding the fossil diversity from the Caturrita Formation. Similarly, whereas lagerpetids are recorded from the Santa Maria Formation strata (Cabreira et al., 2016; Garcia et al., 2019), the group was not recorded for the Caturrita Formation. Both hypotheses (early-diverging crocodylomorph or lagerpetid affinities) imply the increase of the Caturrita Formation diversity. It is important to note that these anatomical traits are not necessarily indicative of affinities with these aforementioned clades. Archosauromorphs experienced diverse episodes of convergence during their evolutionary history and produced a wide range of enigmatic taxa during the Triassic Period (Stocker et al., 2016; Sengupta, Ezcurra & Bandyopadhyay, 2017; Ezcurra et al., 2020; Nesbitt et al., 2021; Yáñez et al., 2021). Based on the phylogenetic analysis performed here, *Faxinalipterus* is nested within the Lagerpetidae.

### Maehary bonapartei

Whereas CAPPA/UFSM 0300 bears a unique suit of traits, the maxillary and dental morphology resemble that of UFRGS-PV-0927-T. As exposed above (see also “Taphonomic Remarks” in the Supplemental Information), the assignment of UFRGS-PV-0927-T to *Faxinalipterus minimus* is unsupported (*e.g.*, UFRGS-PV-0927-T and the holotype where not excavated together and there are no overlapping bones between the specimens). As a consequence, UFRGS-PV-0927-T is taxonomically disassociated from *Faxinalipterus minimus*. Similarly, CAPPA/UFSM 0300 and the holotype of *Faxinalipterus minimus* lack overlapping bones. Therefore, the assignment of CAPPA/UFSM 0300 to *Faxinalipterus minimus* is impracticable. Although plausible, it demands additional specimens with overlapping bones in order to confirm its unique anatomy. At this point, the anatomy of CAPPA/UFSM 0300 is not shared with any valid taxon, providing support to the new taxonomic proposal. Whereas UFRGS-PV-0927-T cannot be referred to *Faxinalipterus minimus*, its morphology resembles CAPPA/UFSM 0300. Both specimens share a gracile maxilla with a rostrocaudally narrow dorsal process and an antorbital fossa restricted to the dorsal process and a wide antorbital fenestra. In addition, the teeth of UFRGS-PV-0927-T are cylindrical, pointed and without serrations, as in CAPPA/UFSM 0300. The presence of the apicobasal sulci running on the mesial and distal margins of the labial surface of the teeth of UFRGS-PV-0927-T is uncertain given its poor preservation. One slight difference between the specimens relies on the robustness of the dorsal process, which is larger in CAPPA/UFSM 0300. It is known that proportions of distinct portions of the maxilla are variable through ontogenetic development in archosaurs (*e.g.*, Bhullar et al., 2012; Fabbri et al., 2021). So, the difference between the specimens may represent intraspecific variation, being more plausible to recognize UFRGS-PV-0927-T as an additional specimen of *Maehary bonapartei* instead of a different taxon with a close peculiar morphology and from the same fossiliferous locality.

**Figure 9:**
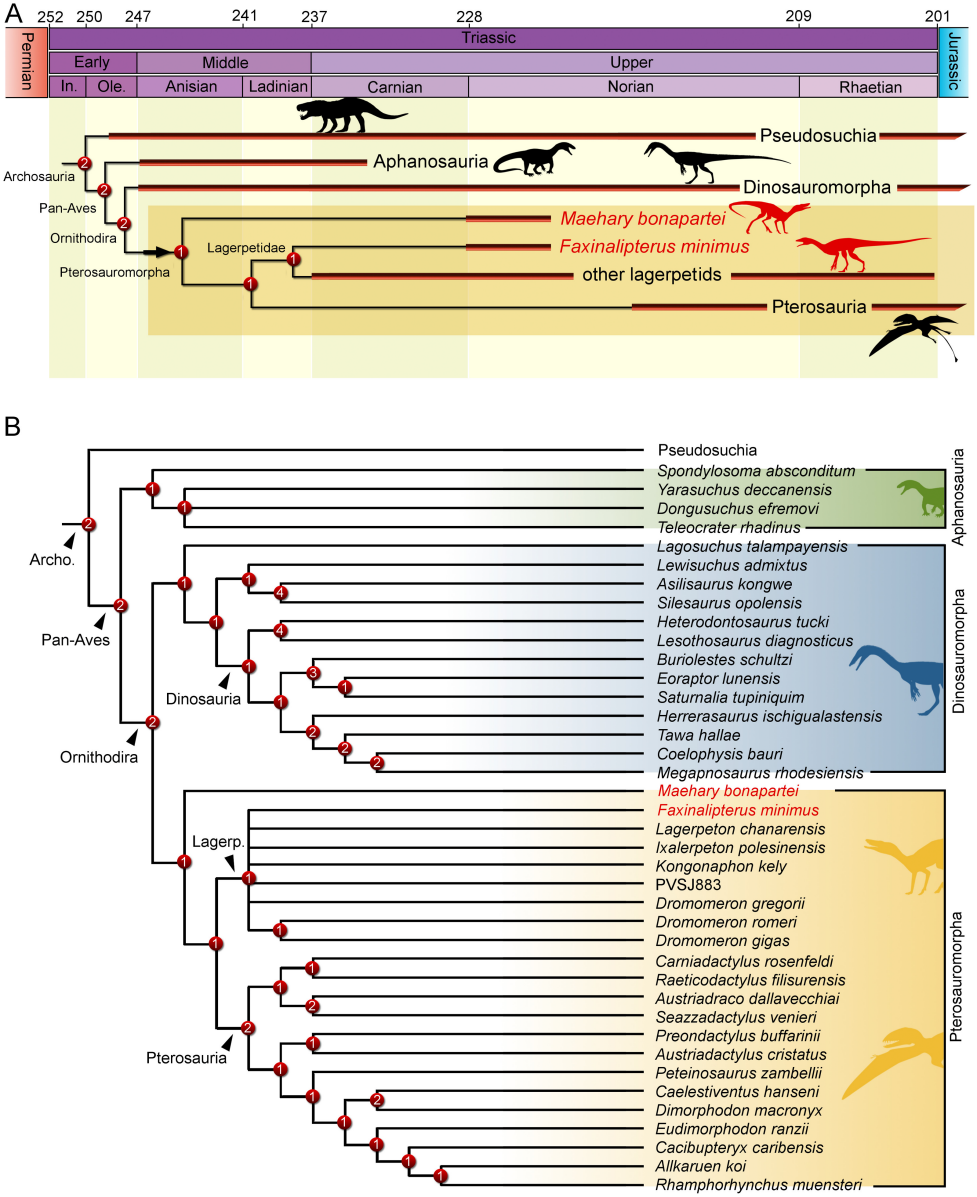
Results of the phylogenetic analysis depicting the position of *Faxinalipterus minimus* and *Maehary bonapartei* gen. et sp. nov. (A) Time-calibrated reduced strict consensus tree. (B) Reduced strict consensus tree. Archo., Archosauria; Lagerp., Lagerpetidae. Numbers on nodes represent Bremer support values. Silhouettes based on the artwork by Márcio L. Castro, Rodolfo Nogueira and Corey Ford.

*Maehary bonapartei* represents a peculiar archosaur with putative pterosauromorph affinities. However, the new taxon bears several traits that are unusual for pterosauromorphs (*i.e.*, pterosaurs and lagerpetids), such as an expanded antorbital fossa, an elongated rostroventral process of the nasal, and a rounded rostral end of the dentary. Its unique morphology expands the disparity of archosaurs during the Late Triassic and sheds light on the early branches of the pterosauromorph radiation. The dental anatomy (*e.g.*, conical teeth lacking serrations) and body size of *Maehary bonapartei* provides evidence on additional niche occupation by small archosaurs. Despite the shared absence of serrations, the new species lacks heterodonty and multi-cusped teeth (Fig. 7K), differing from the typical condition of early pterosaurs (Dalla Vecchia, 2013). Likewise, the new species lacks the small accessory cusps present in lagerpetids (Ezcurra et al., 2020). According to the phylogenetic position of *Maehary bonapartei* (Fig. 9), the absence of serrations was the ancestral condition of lagerpetids and pterosaurs, whereas the cusps evolved during a second step within the evolutionary history of pterosauromorphs. Alternatively, the presence of dental cusps could have evolved earlier, being lost in *Maehary bonapartei*.

## Conclusions

Additional mechanical preparation provided new anatomical information on the holotype of *Faxinalipterus minimus*. Several bones were reinterpreted and reexamined, differing from the identifications provided by Bonaparte, Schultz & Soares (2010). We concluded that the holotype of *Faxinalipterus minimus* fails to show any pterosaur traits. The specimen carries a unique combination of traits among archosaurs, maintaining its status as a valid genus and species. The phylogenetic analysis performed here recovered *Faxinalipterus minimus* in a polytomy within Lagerpetidae. The partial maxilla originally ascribed to the taxon is disassociated from *Faxinalipterus minimus* and referred to *Maehary bonapartei*, a new taxon described here from a partial skull with lower jaw and a handful of postcranial elements. *Maehary bonapartei* bears a peculiar anatomy and is here regarded as an early-diverging pterosauromorph.

## Supplemental Information

10.7717/peerj.13276/supp-1 Supplemental Information 1 Taphonomic Remarks

10.7717/peerj.13276/supp-2 Supplemental Information 2 Data Matrix of the phylogenetic analysis

## Institutional Abbreviations

CAPPA/UFSM: Centro de Apoio à Pesquisa Paleontológica da Quarta Colônia da Universidade Federal de Santa Maria, Santa Maria, Rio Grande do Sul, Brazil
UFRGS-PV: Paleovertebrate Collection of the Universidade Federal do Rio Grande do Sul, Rio Grande do Sul, Brazil.

## Additional Information and Declarations

### Data Availability

The following information was supplied regarding data availability: The scan and the 3D models are available at Morphosource: https://www.morphosource.org/projects/000392934?locale=en. DOI: 10.17602/M2/M393066.

### New Species Registration

The following information was supplied regarding the registration of a newly described species: Publication LSID: urn:lsid:zoobank.org:pub:8C6EAD24-B978-45C3-A3BB-71D2F2E8E48F Genus Maehary LSID: urn:lsid:zoobank.org:act:81652416-407D-4966-B3A9-7EF2EE3E3171. Species Maehary bonapartei LSID: urn:lsid:zoobank.org:act:5576C87F-853B-4BD2-8D9D-DF7A174F3B06.
