# Supplementary material for: Reassessment of Faxinalipterus minimus, a purported Triassic pterosaur from southern Brazil with the description of a new taxon
Source: PeerJ. 2022 May 3;10:e13276. doi: 10.7717/peerj.13276 (PMC9074864; doi:10.7717/peerj.13276)
Supplement: Supplemental Information 1 [file peerj-10-13276-s001.docx]

**Taphonomic Remarks**

The faunal content of the massive sandstone facies of the Linha São Luiz Site is exclusively composed of terrestrial vertebrates. The only large-sized animal (ca. 2 m length) found at these beds is the dinosaur *Guaibasaurus candelariensis* (*Bonaparte et al., 1999*). It draws attention the almost exclusive occurrence of small-sized tetrapods (most of them not reaching the size of the extant rat *Rattus norvegicus*), such as the procolophonian *Soturnia caliodon* (*Cisneros & Schultz, 2003*); the basal lepidosauromorph *Cargninia enigmatica* (*Bonaparte et al., 2010; Romo de Vivar et al., 2020a*); the sphenodontians *Clevosaurus brasiliensis*, *Lanceirosphenodon ferigoloi* (*Bonaparte & Sues, 2006; Romo de Vivar et al., 2020b*) and *Microsphenodon bonapartei* (*Chambi-Trowell et al., 2021*); and the non-mammaliaform probainognathian cynodonts *Brasilodon quadrangularis*, *Riograndia guaibensis* and *Irajatherium hernandezi* (*Bonaparte et al., 2001, 2003, 2005; Martinelli et al., 2005; Soares et al., 2011; Oliveira et al., 2011; Guignard et al., 2019a, 2019b; Kerber et al., 2021a; 2021b*), aside from the specimens we describe in the manuscript “Reassessment of *Faxinalipterus minimus* minimus, a purported Triassic pterosaur from southern Brazil with the description of a new taxon”.

The small fossil tetrapods (which currently compose a sample of more than a hundred specimens) are preserved as semi-articulated skeletons (less frequently) or isolated bones, those being represented mostly by skulls and lower jaws. *Horn et al. (2018)* suggested this kind of accumulation could have had as a concentration agent a large carnivore predator with preference for small prey, disposing carcasses in a small area. As skulls and lower jaws are the less nutritional body parts, they were left as scrap (*Behrensmeyer, 1991; Rogers & Broughton, 2001*). These remaining, unconsumed parts of the skeletons were then subjected to subaerial exposure being gradually accumulated while underwenting biostratinomic processes (e.g., disarticulation, weathering, trampling) before their final burial by ephemeral floods.

According to *Horn et al. (2018),* the sedimentary environment reveals highly variable depositional rates and periodicity within the massive channel facies, with possibly long periods of non-deposition. This would enable a mix of well-preserved skulls and lower jaws, plus some disarticulated postcranial bones being buried together in each flood event.

**References**

**Behrensmeyer AK**. 1991. Terrestrial vertebrate accumulations. In: Allison, PA, Briggs, DE (eds.). Taphonomy: releasing the data locked in the fossil record. Plenum Press New York, 9:291-335.

**Bonaparte JF, Ferigolo J, Ribeiro AM.** 1999. A new Early Late Triassic saurischian dinosaur from Rio Grande do Sul State, Brazil. National Sciences Museum Monographs 15: 89-109.

**Bonaparte JF, Ferigolo J, Ribeiro AM**. 2001. A primitive Late Triassic “ictidosaur” from Rio Grande do Sul, Brazil. Palaeontology 44: 623-635.

**Bonaparte JF, Martinelli AG, Schultz CL, Rubert R.** 2003. The sister group of mammals: small cynodonts from the Late Triassic of southern Brazil. Revista Brasileira de Paleontologia 5: 5–27.

**Bonaparte JF, Martinelli AG, Schultz CL.** 2005. New information on *Brasilodon* and *Brasilitherium* (Cynodontia, Probainognathia) from the Late Triassic of southern Brazil. Revista Brasileira de Paleontologia 8: 25-46.

**Bonaparte JF, Sues HD**. 2006. A new species of *Clevosaurus* (Lepidosauria: Rhynchocephalia) from the upper Triassic of Rio Grande do Sul, Brazil. Palaeontology, 49(4): 917-923.

**Bonaparte JF, Schultz CL, Soares MB, Martinelli AG.** 2010. La fauna local de Faxinal do Soturno, Triásico Tardío de Rio Grande do Sul, Brasil. Revista Brasileira de Paleontologia 13:233–246.

**Bonaparte JF, Schultz CL, Soares. MB.** 2010b. Pterosauria from the late Triassic of Southern Brazil; pp. 63-71 in Bandyopadhyay S (ed.), New Aspects of Mesozoic Biodiversity, Lecture Notes in Earth Sciences 132.

**Guignard ML, Martinelli AG, Soares MB.** 2019a. Postcranial anatomy of *Riograndia guaibensis* (Cynodontia: Ictidosauria). Geobios 53, 9–21.

**Guignard ML, Martinelli AG, Soares MB.** 2019b. The postcranial anatomy of *Brasilodon quadrangularis* and the acquisition of mammaliaform traits among nonmammaliaform cynodonts. PloS One 14 (5), e0216672.

**Horn BLD, Goldberg K, Schultz CL.** 2018. A loess deposit in the Late Triassic of southern Gondwana, and its significance to global paleoclimate. Journal of South American Earth Sciences 81: 189–203.

**Kerber L, Ferreira JD, Fonseca PHM, Franco A, Martinelli AG, Soares MB, Ribeiro AM**. 2021a. An additional brain endocast of the ictidosaur *Riograndia guaibensis* (Eucynodontia: Probainognathia): intraspecific variation of endocranial traits. An Acad Bras Cienc 93: e20200084.

**Kerber L, Martinelli AG, Müller RT, Pretto FA.** 2021b. A new specimen provides insights into the anatomy of *Irajatherium hernandezi*, a poorly known probainognathian cynodont from the Late Triassic of southern Brazil. The Anatomical Record. 10.1002/ar.24830.

**Martinelli AG, Bonaparte JF, Schultz CL, Rubert, R.** 2005. A new tritheledontid (Therapsida, Eucynodontia) from the Late Triassic of Rio Grande do Sul (Brazil) and its phylogenetic relationships among carnivorous nonmammalian eucynodonts. Ameghiniana 42: 191–208.

**Martinelli AG, Escobar JA, Francischini H, Kerber L, Müller RT, Rubert R, Schultz CL, Da-Rosa AAS.** 2020. New record of a stahleckeriid dicynodont (Therapsida, Dicynodontia) from the Late Triassic of southern Brazil and biostratigraphic remarks on the Riograndia Assemblage Zone. Historical Biology Online first: 1–10.

**Oliveira TV, Martinelli AG, Soares MB.** 2011. New information about *Irajatherium hernandezi* Martinelli, Bonaparte, Schultz & Rubert 2005 (Eucynodontia, Tritheledontidae) from the upper triassic (Caturrita Formation, Paraná Basin) of Brazil. PalZ 1(85): 67–82.

**Rogers AR, Broughton, JM.** 2001. Selective transport of animal parts by ancient hunters: a new statistical method and an application to the Emeryville Shellmound Fauna. Journal of Archaeological Science, 28: 763-773.

**Romo de Vivar PR, Martinelli AG, Fonseca PM, Soares MB**. 2020a. To be or not to be: the hidden side of Cargninia enigmatica and other puzzling remains of Lepidosauromorpha from the Upper Triassic of Brazil. Journal of Vertebrate Paleontology, e1828438.

**Silva RC, Barboni R, Dutra T, Godoy MM, Binotto RB.** 2012. Footprints of large theropod dinosaurs and implications on the age of Triassic biotas from Southern Brazil. Journal of South American Earth Sciences 39: 16–23.

**Soares MB, Schultz CL, Horn BLD.** 2011. New information on *Riograndia guaibensis* Bonaparte, Ferigolo & Ribeiro, 2001 (Eucynodontia, Tritheledontidae) from the Late Triassic of southern Brazil: anatomical and biostratigraphic implications. Anais da Academia Brasileira de Ciências 83: 329–354.
